# A Case of Raynaud's Disease
*Read (and the patient shown) before the Bristol Medico-Chirurgical Society, April, 1888.


**Published:** 1888-12

**Authors:** Henry Waldo

**Affiliations:** Physician to the Bristol Royal Infirmary, and in charge of the Department for Skin Diseases


### A CASE OF RAYNAUD'S DISEASE.* By HENRY WALDO, M.D., M.R.C.P.; Physician to the Bristol Royal Infirmary, and in charge of the Department for Skin Diseases.

E. D., æt. 29, single. A general servant up to within three months. Admitted to the Infirmary 18th April, 1888. Father alive and well. Mother died sixteen years

* Read (and the patient shown) before the Bristol Medico-Chirurgical Society, April, 1888.

ago. Has one brother, and he is well. Lost one sister and one brother when they were babies.

Usual health very good, with the exception of what she calls a bilious attack, in which she gets much headache and vomiting, and visions move before her eyes and the sight goes. These attacks are less frequent than they used to be. No audible cardiac murmur. Arterial tension somewhat increased. Is subject to attacks of epistaxis. The knee-jerks are equal and exaggerated. She has noticed that her toes have been dark-coloured, and cold and sore for the last three or four years. They are worse in winter. There is also discolouration of hands and wrist, and of the face. Her feet and hands often get numb and cold: she has no feeling in them the first thing in the morning. She is subject to paroxysms of agonising pain, which occur on and off for a week at a time, and so severe is it that she is obliged to get up and walk about her room at night. She can walk well. Sensibility normal. Sight good. Urine 1024, acid, no albumen or sugar. Catamenia regular, and rather profuse. Tongue fairly clean. Bowels regular.

Raynaud first called attention to this condition as a very remarkable neurosis, and showed its alliance with the well-recognised, trivial, every-day complaint of numb or dead fingers. Young women and emotional persons are sometimes subject to their fingers going dead. Exposure to cold, taking a cold bath before breakfast, staying too long in the sea, anything which shocks or depresses the circulation, determines the event in certain individuals. Now if this state of the circulation is much prolonged, or if, from some further abnormality in the blood itself, the capillary nutritional interchanges are rendered impracticable, the part thus starved of blood does not reanimate, but dies. Raynaud called this local syncope. A more serious state of affairs he called local asphyxia. Here the complete contraction of the arterioles does not blanch the vascular area; the part, a finger-tip perhaps, instead of paling, becomes livid, slate-coloured, purplish or black, stasis of blood, venous congestion—part numb, cold, lost tactile sense and local sensibility (anæsthetic and analgesic). The affected part does not bleed on pricking, and there is intense burning pain, only relieved by plunging the fingers into cold water. In local syncope there is complete absence of blood, and much cold. In local asphyxia there is some circulation of venous blood, and retention of heat. There is always a tendency to symmetry in these cases—upper or lower extremities, both ears, median parts, as tip of nose or over the coccyx.

Raynaud considered this disease a neurosis in which the normal excito-motor function of the spinal cord by which it presides over the vaso-motor nerves is exaggerated, and there occur cramp-like contractions of the arterio-capillaries, and to this is due the attacks of pain which is such a prominent symptom in these cases.

In the case I have shown you, in addition to discolouration of the extremities, the patient is subject to attacks of megrim, which is undoubtedly a neurosis; and I see Latham considers the pathological cause of megrim to be some affection of the vaso-motor nerves, in virtue of which the vessels become contracted and the tissues anæmic.

Vulpian, in 1875, described symmetrical congestion of the extremities observed in certain cases of neuralgia.

Billroth, in 1878, recorded a case of discolouration of finger-tips, followed by gangrene and necrosis of phalanges resembling paronychia, and terminating in exfoliation of small, dry, parchment-like crusts, of two years' duration.

The female sex seem to be preferentially affected with this disease, and it occurs mostly between the ages of 18 and 30. It is sometimes associated with diabetes.

Colcott Fox says, this year, that experience has shown him that sclerodermic patients with the extremities affected are very liable to suffer from attacks of Raynaud's disease of the fingers and toes. He also says that there is yet another class of cases in which congestive patches occur over the body (with or without urine changes), of all degrees of severity—evanescent, like urticaria patches, or superficially gangrenous.

This patient has suffered with what she calls chilblains of the upper part of the pinna of each ear—that part of the ear which seems to be least well supplied with blood, and it is the part affected most frequently in association with lupus erythematosus of the face and fingers—a disease which is thought to depend upon a faulty supply of blood to these parts.

In conclusion, I may say that many other skin-diseases undoubtedly depend upon a neurosis, and it is probable that many cases of pemphigus, herpes, and other cutaneous diseases are caused by a peripheral neuritis of sensory nerves.

Raynaud advises as drugs quinine and strychnia; also constant descending electrical currents down the spine. Barlow insists upon systematic daily *local* galvanising, rather than through the spine. Warm clothing and shampooing is also important in the treatment.*

* While this has been passing through the press, the patient has been readmitted to the Infirmary with gangrene of the finger-tips.

